# Crosslinked Graft Copolymer of Methacrylic Acid and Gelatin as a Novel Hydrogel with pH-Responsiveness Properties

**DOI:** 10.3390/ma4030543

**Published:** 2011-03-02

**Authors:** Mohammad Sadeghi, Behrouz Heidari

**Affiliations:** 1 Department of Chemistry, Science Faculty, Islamic Azad University, Arak Branch, 38156 Arak, Iran; 2 Department of Chemical Engineering, Engineer Faculty, Islamic Azad University, Arak Branch, 38156 Arak, Iran; E-Mail: Heidari@yahoo.com

**Keywords:** gelatin, methacrylic acid, graft copolymers

## Abstract

In this paper, a novel gelatin-based hydrogel was synthesized through crosslinking graft copolymerization of methacrylic acid (MAA) onto gelatin, using ammonium persulfate (APS) as a free radical initiator in the presence of methylenebisacrylamide (MBA) as a crosslinker. A proposed mechanism for hydrogel formation was suggested and the structure of the product was established using FTIR spectroscopy and gravimetric analysis of the products. Moreover, morphology of the samples was examined by scanning electron microscopy (SEM) and thermogravimetric analysis (TGA/DTG). The effect of reaction variables such as concentration of APS and MBA were systematically optimized to achieve a hydrogel with swelling capacity as high as possible. The gelatin-g-PMAA hydrogel exhibited a pH-responsiveness character so that a swelling-deswelling pulsatile behavior was recorded at pHs 2 and 8. This on-off switching behavior makes the hydrogel as a good candidate for controlled delivery of bioactive agents.

## 1. Introduction

Synthesis and characterization of hydrogels is the main goal of the several research groups in the world. These materials are defined as hydrophilic, three-dimensional networks with ability to absorb large values of water, saline solution, or physiological fluids [1]. They are widely used in various applications such as hygienics, foods, cosmetics, and agriculture. Nowadays, the worldwide production of SAP(hydrogel) is more than one million tons in year.

The properties of the swelling medium (e.g., pH, ionic strength and the counter ion and its valency) affect the swelling characteristics. SAPs responding to external stimuli such as heat, pH, electric field, chemical environments, etc., are often referred to as "*intelligent*" or "*smart*" polymers. Among these, pH-sensitive hydrogels have been extensively investigated for potential use in site-specific delivery of drugs to specific regions of the gastrointestinal tract and have been prepared for delivery of low molecular weight protein drugs. Therefore, these hydrogels have important applications in the fields of medicine, pharmacy, and biotechnology [2,3].

Natural-based hydrogels have attracted much interest from the viewpoint of improving the tissue tolerance of synthetic polymers and the mechanical properties of natural polymers. The presence of the natural parts guarantees biodegradability of the superabsorbing materials. Because of their bio Compatibility, biodegradability and non-toxicity, natural polymers, i.e., polysaccharides and proteins, are the main part of these biopolymers. One of the best methods for the synthesis of these hydrogels is graft copolymerization of vinylic monomers onto natural polymers. Monomers such as acrylonitrile (AN), acrylic acid (AA), acrylamide (AAm) have been graft copolymerized onto polysaccharides such as starch, cellulose and their derivatives [4,5,6,7]. The first industrial hydrogel was synthesized using this method via ceric-induced graft copolymerization of acrylonitrile onto starch followed by alkaline hydrolysis of the resulted graft copolymer [8].

Proteins are widely distributed in nature and are synthesized mainly in animals, i.e., collagen, keratin, gelatin, and etc., and in a few plants such as Soya. In general, proteins are high molecular weight polymers and their solubility in aqueous solutions is difficult. Two efficient methods for preparation of aqueous soluble proteins are alkaline and enzymatic hydrolysis.

In the present report, to modify the hydrolyzed gelatin, the grafting of metacrylic acid (MAA) onto gelatin chains in the presence of a crosslinking agent was performed in a homogeneous system.

## 2. Results and Discussion

### 2.1. Synthesis of Hydrogels

**Scheme 1:**
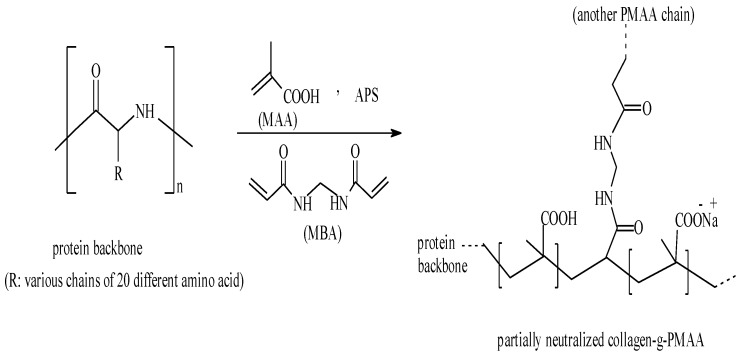
Proposed mechanistic pathway for synthesis of the gelatin-*g*-PMAA hydrogel.

A general reaction mechanism for crosslinking graft copolymerization of MAA onto gelatin backbones in the presence of APS and MBA is shown in Scheme 1. The sulfate anion-radical produced from thermaly decomposition of APS, abstracts hydrogen from one of the functional groups in side chains (*i.e*., COOH, SH, OH, and NH_2_) of the substrate to form the corresponding radical [9]. Then the resulted macroradicals radically initiate graft copolymerization of neutralized MAA led to a graft copolymer socalled gelatin-g-PMAA. Since a crosslinking agent, *i.e*., MBA, presented in the reaction mixture, the crosslinked gelatin-g-PMAA network is resulted.

### 2.2. Optimization of the Grafting Variables

In this work, the main factors affecting on the grafting conditions (*i.e*., concentration of MBA and APS) as well as the swelling behavior of the resuled pH-responsive hydrogels were investigated.

#### 2.2.1. Effect of MBA Concentration

**Figure 1:**
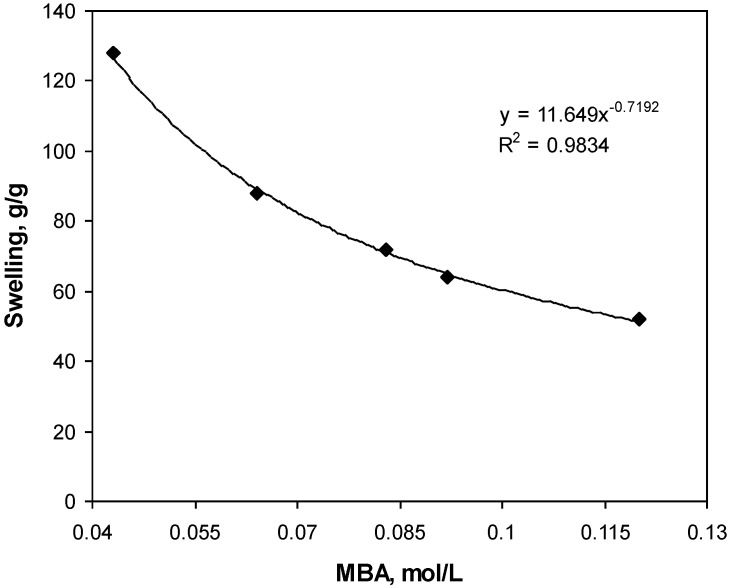
Effect of crosslinker concentration on swelling capacity.

Figure 1 shows the influence of the crosslinking agent on the swelling capacity of gelatin-g-PMAA hydrogel. Higher values of absorbency is obtained using lower crosslinker concentration (Cc), however, the hydrogels prepared do not posses good dimensional stability, so that the swollen gel strength is not sufficient to be referred as a real. In fact, with Cc 0.043 mol/L, slimy gel formed. Figure 1 exhibits a power law behavior of absorbency-Cc. Such a behavior is well-known, as reported by pioneering scientists [10]. Higher crosslinker concentration decreases the space between the copolymer chains and, consequently, the resulted highly crosslinked rigid structure cannot be expanded and hold a large quantity of water [11].

#### 2.2.2. Effect of APS Concentration

**Figure 2:**
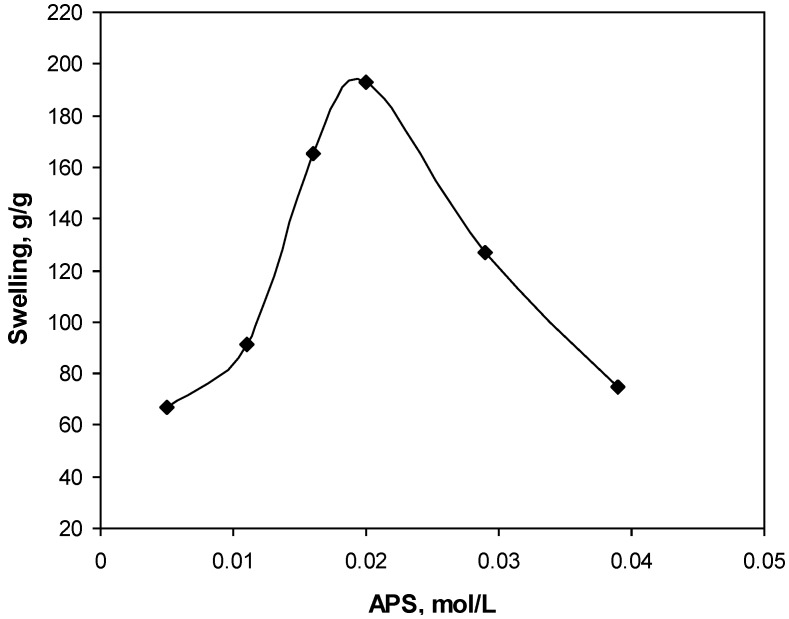
Effect of initiator concentration on swelling capacity.

The relationship between the initiator concentration and water absorbency values was studied by varying the APS concentration from 0.001 to 0.04 mol/L (Figure 2). It is observed that the absorbency is substantially increased with increasing in the APS concentration and then it is decreased. Initial increment in water absorbency may be attributed to increased number of active free radicals on the gelatin backbone. Subsequent decrease in swelling is originated from an increase in terminating step reaction via bimolecular collision, which, in turn, causes to enhance crosslinking density. This possible phenomenon is referred to as “self crosslinking” by Chen and Zhao [12]. In addition, the free radical degradation of gelatin backbones by sulfate radical-anions is an additional reason for swelling-loss at higher APS concentration. The proposed mechanism for this possibility is reported in the previous work [13]. A similar observation is reported by Hsu et al. in the case of degradation of chitosan with potassium persulfate [14].

### 2.3. Spectral Characterization

**Figure 3:**
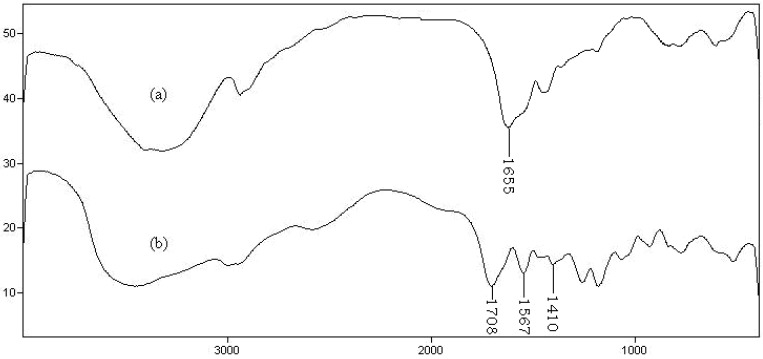
FTIR spectra of hydrolyzed gelatin **(a)** and gelatin-g-PMAA hydrogel **(b)**.

For identification of the hydrogel, infrared spectroscopy was used. Figure 3 shows the FTIR spectra of the hydrolyzed Gelatin and the synthesized hydrogel. The band observed at 1655 cm^−1^ can be attributed to C=O stretching in carboxamide functional groups of substrate backbone (Figure 3a). The broad band at 3200–3600 cm^−1^ is due to stretching of –OH groups of the gelatin. The IR spectrum of the hydrogel, gelatin-g-PMAA (Figure 3b) shows three new characteristic absorption bands at 1708, 1567 and 1410 cm^−1^ verifying the formation of graft copolymer product. These peaks attributed to carbonyl stretching of the carboxylic acid groups and symmetric and asymmetric stretching modes of carboxylate anions, respectively. Combination of absorption of the carboxylate and alcoholic O–H stretching bands is appeared in the wide range of 2550–3600 cm^−1^.

Transmittance/Wavenumber(cm^−1^)

To obtain additional evidence of grafting, a similar polymerization was conducted in the absence of the crosslinker. After extracting the homopolymers, PMAA and unreacted monomers using a cellophane membrane dialysis bag (D9402, Sigma–Aldrich), an appreciable amount of grafted gelatin (85%) was observed. The graft copolymer spectrum was very similar to Figure 3b. Also according to preliminary measurements, the sol (soluble) content of the hydrogel networks was as little as 1.2%. This fact practically proves that all monomers are involved in the polymer network. So, the monomers percent in the network will be very similar to that of the initial feed of reaction.

### 2.4. Scanning Electron Microscopy

**Figure 4:**
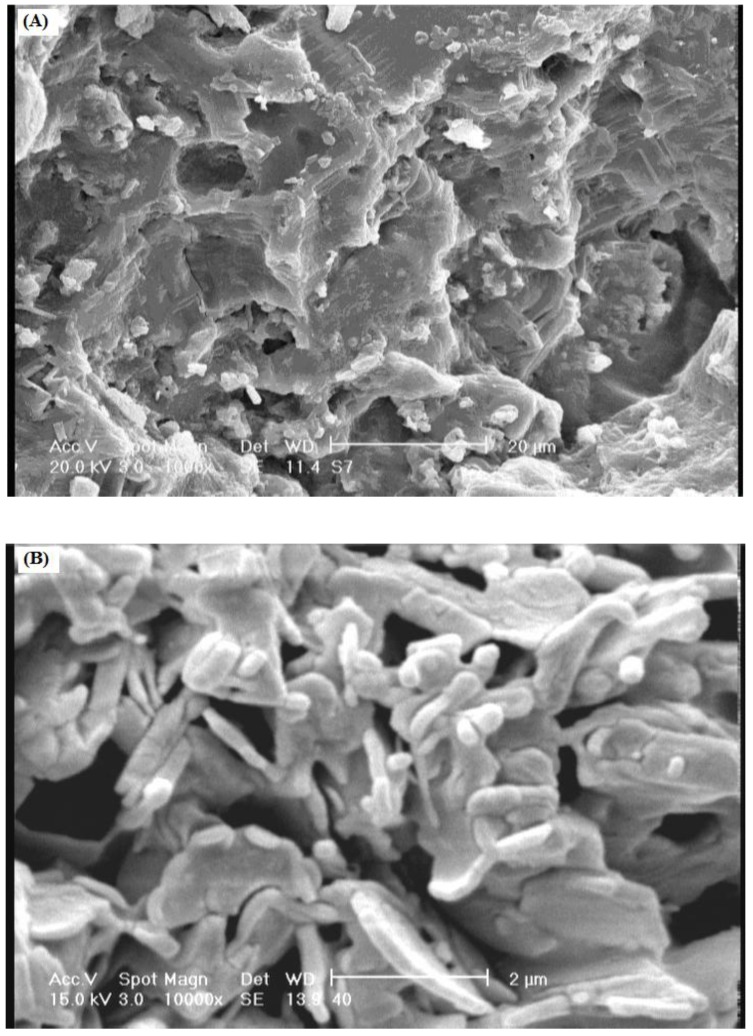
SEM photograph of the optimized hydrogel (gelatin 1.5 g, MBA 0.043 mol/L, APS 0.02 mol/L, 55 °C, 60 min). **(A)** Surface of porous hydrogel; **(B)** Cross-sectional area of porous hydrogel.

One of the most important properties that must be considered is hydrogel microstructure morphologies. Figure 4 shows the scanning electron microscope (SEM) photographs of the surface (Figure 4A) and the cross-sectional area (Figure 4B) of the hydrogel with interconnected pores. These pictures verify that the synthesized polymer in this work have a porous structure, where the pores might be induced in to the hydrogel by water evaporation resulting from reaction heat. It is supposed that these pores are the regions of water permeation and interaction sites of external stimuli with the hydrophilic groups of the graft copolymers. The cross-sectional view of hydrogels (Figure 4B) also exhibited a large, open, channel-like structure.

The porosity plays the multiple role of enhancing the total water sorption capability and the rate of response by reducing the transport resistance. Therefore, creation of porosity in hydrogels has been considered as an important process in many ways. The phase-separation technique, the water-soluble porogens and the foaming technique are three different methods for preparing porous hydrogel structures. In this paper, as mentioned above, however the pores were simply produced from water evaporation resulting from reaction medium heat.

### 2.5. Thermogravimetric Behavior

**Figure 5:**
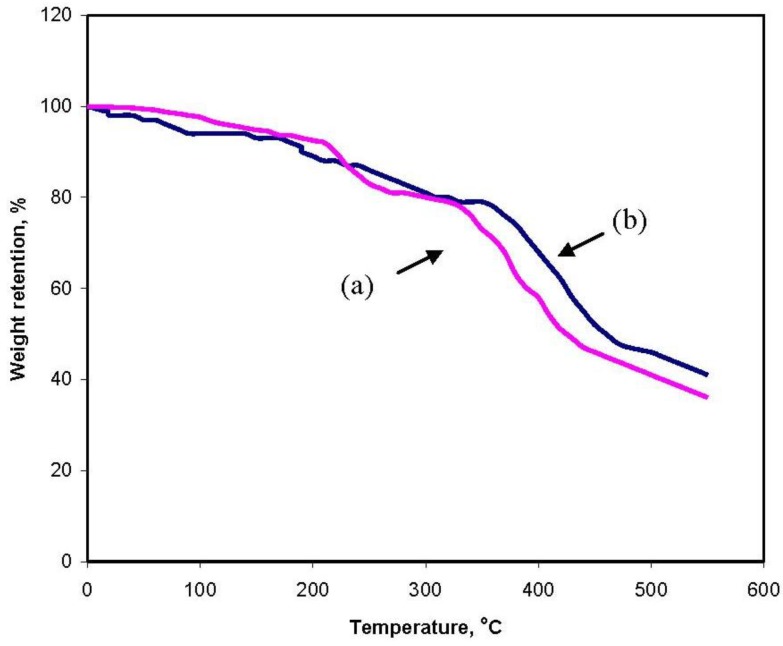
TGA curves of **(a)** gelatin and **(b)** gelatin-*g*-PMAA.

The grafting was also supported by thermogravimetric analysis (Figure 5). TGA of gelatin (Figure 5a) shows a weight loss in two distinct stages. The first stage ranges between 15 and 120 °C and shows about 17% loss in weight. This may correspond to the loss of adsorbed and bound water. No such inflexion was observed in the TGA curve of gelatin-g-PMAA. The second stage of weight loss starts at 330 °C and continues up to 440 °C during which there was 60% weight loss due to the degradation of gelatin. Grafted samples, however, show almost different behavior of weight loss between 15 and 550 °C (Figure 5b). The first stage of weight loss starts at 205 °C and continues up to 330 °C due to the degradation of gelatin. The second stage from 370 to 480 °C may contribute to the decomposition of different structure of the graft copolymer. The appearance of these stages indicates the structure of gelatin chains has been changed, which might be due to the grafting of PMAA chains. In general, the copolymer had lower weight loss than gelatin. This means that the grafting of gelatin increases the thermal stability of gelatin in some extent.

**Figure 6:**
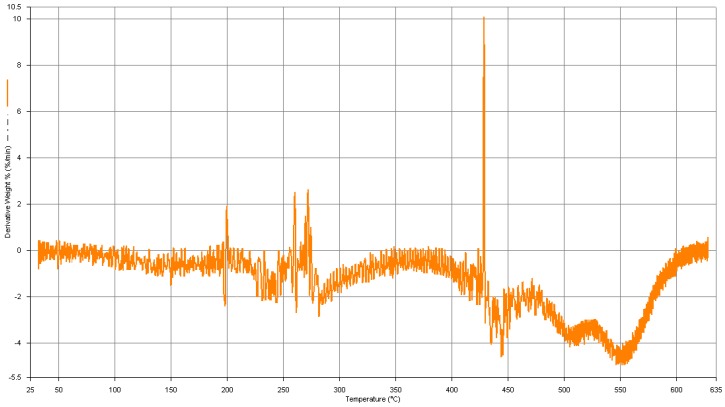
DTG curve of optimized hydrogel.

For the better study of the thermal behavior of the hydrogel, curve of the hydrogel was also provided as shown in Figure 6. The first derivative of the TGA curve (DTG) shows that the maximum decomposition rate of the hydrogel occurs in the sharp peak at 432 °C. At this temperature, an endothermic reaction cause to decomposition of the hydrogel. Other main decomposition points of the hydrogel are at 198, 263, and 274 °C and all are endothermic decompositions.

### 2.6. pH-Responsiveness Behavior of Gelatin -g-PMAA Hydrogel

**Figure 7:**
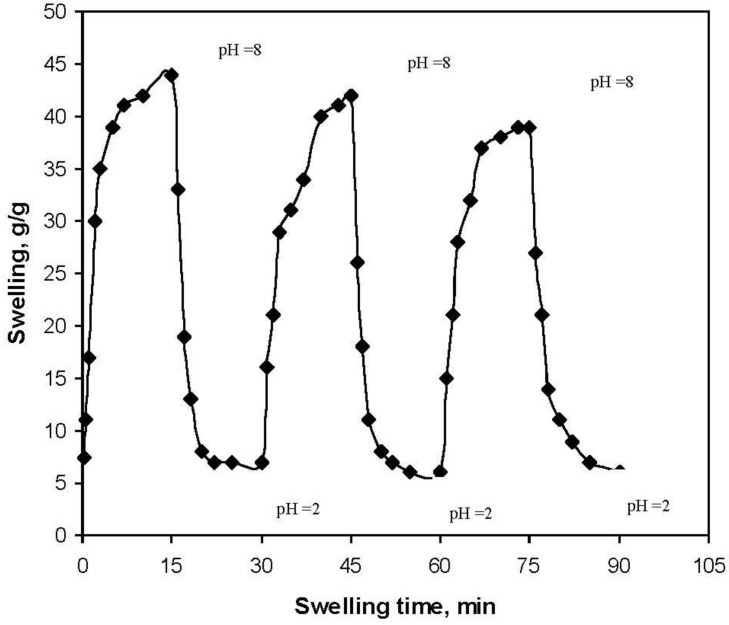
On-off switching behavior as reversible pulsatile swelling (pH 8.0) and deswelling (pH 2.0) of the gelatin-g-PMAA hydrogel.

We investigated the reversible swelling-deswelling behavior of this hydrogel in solutions with pH 2.0 and 8.0 (Figure 7). At pH 8.0, the hydrogel swells due to anion-anion repulsive electrostatic forces, while at pH 2.0, it shrinks within a few minutes due to protonation of the carboxylate anions. This swelling-deswelling behavior of the hydrogels makes them as suitable candidates for designing drug delivery systems.

## 3. Experimental

### 3.1. Materials

Gelatin (Merck) was used as received. Acrylic acid (AA, Merck) was used after vacuum distillation. N’,N’-methylene bisacrylamide and ammonium persulfate (Fluka) were of analytical grade and used without further purification. Double distilled water was used for the hydrogel preparation and swelling measurements.

### 3.2. Preparation of Hydrogel

Gelatin (1.50 g) was dissolved in 35 mL distilled water and filtered to remove its insoluble salt. Then the solution was added to a three-neck reactor equipped with a mechanical stirrer. The reactor was immersed in a thermostated water bath preset at a desired temperature (55 °C). Then the initiator solution (0.01–0.40 g) was added to the mixture. After stirring for 10 min, certain amounts of 70% neutralized MAA (2.0–8.0 g) and MBA (0.05–0.20 g) were simultaneously added to the reaction mixture. After 60 min, the produced hydrogel was poured to excess non-solvent ethanol (200 mL) and remained for 3 h to dewater. Again, 100 mL fresh ethanol was added and the hydrogel was remained for 24 h. Finally, the filtered hydrogel is dried in oven at 60 °C for 10 h.

### 3.3. Absorbency at Various pHs 

Sensitivity of the hydrogel to pH was investigated in terms of swelling and deswelling of the final product at two basic (pH 7.0) and acidic (pH 2.0) solutions, respectively. The pH values were precisely checked by a pH-meter (Metrohm/620, accuracy ±0.1). Swelling capacity of the hydrogels at each pH was measured according to a conventional tea bag method at consecutive time intervals (15 min).

### 3.4. Instrumental Analysis 

Fourier transform infrared (FTIR) spectroscopy absorption spectra of samples were taken in KBr pellets, using an ABB Bomem MB-100 FTIR spectrophotometer (Quebec, Canada), at room temperature. To study the morphology of the hydrogel, the surface and cross-sectioned area of the hydrogel were examined using scanning electron microscopy (SEM). After Soxhlet extraction with methanol for 24 h and drying in an oven, powder was coated with a thin layer of gold and imaged in a SEM instrument (Leo, 1455 VP). Thermogravimetric analyses (TGA/DTG) were performed on a Universal V4.1D TA Instruments (SDT Q600) with 8–10 mg samples on a platinum pan under nitrogen atmosphere. Experiments were performed at a heating rate of 20 °C/min until 550 °C.

## 4. Conclusions

The hydrogel, gelatin-g-PMAA, was synthesized by graft copolymerization of methacrylic acid onto gelatin, in a homogeneous medium. The study of FTIR spectra and Thermogravimetric analysis provide the graft copolymerization do takes place. The maximum water absorbency (197 g/g) was achieved under the optimum conditions, that found to be MBA 0.043 mol/L, and APS 0.02 mol/L. The hydrogels exhibited high sensitivity to pH, so that, the reversible swelling-deswelling behavior in solutions with acidic and basic pH makes the hydrogels as a suitable candidate for controlled drug delivery systems.

## References

[1] Buchholz F.L., Graham A.T. (1997). Modern Superabsorbent Polymer Technology.

[2] Flory P.J. (1953). Principles of Polymer Chemistry.

[3] Hoffman A.S., Salamone J.C. (1996). Polymeric Materials Encyclopedia.

[4] Lim D.W., Whang H.S., Yoon K.J. (2001). Synthesis and absorbency of a superabsorbent from sodium starch sulfate-g-polyacrylonitrile. J. Appl. Polym. Sci..

[5] Mahdavinia G.R., Pourjavadi A., Hosseinzadeh H., Zohuriaan M.J. (2004). Modified chitosan 4. Superabsorbent hydrogels from poly(acrylicacid-co-acrylamide)grafted chitosan with salt- and pH-responsiveness properties. Eur. Polym. J..

[6] Athawale V.D., Lele V. (1998). Graft copolymerization onto starch—3: Grafting of acrylamide using ceric ion initiation and preparation of its hydrogels. Starch/Starke.

[7] Sadeghi M., Hosseinzadeh H. (2010). Synthesis and super-swelling behavior of a novel low salt-sensitive gelatin-based superabsorbent hydrogel: collagen-*g*-poly (AMPS). Turk. J. Chem..

[8] Fanta G.F., Salamone J.C. (1996). Polymeric Materials Encyclopedia.

[9] Peppas L.B., Harland R.S. (1990). Absorbent Polymer Technology.

[10] Pourjavadi A., Harzandi A.M., Hosseinzadeh H. (2004). Modified carrageenan. 3. Synthesis of a novel polysaccharide-based superabsorbent hydrogel via graft copolymerization of acrylic acid onto kappa-carrageenan in air. Eur. Polym. J..

[11] Zhang X.Z., Zhuo R.X., Cui J.Z., Zhang J.T. (2002). A novel thermo-responsive drug delivery system with positive controlled release. Int. J. Pharm..

[12] Chen J., Zhao Y. (2000). Relationship between water absorbency and reaction conditions in aqueous solution polymerization of polyacrylate superabsorbents. J. Appl. Polym. Sci..

[13] Sadeghi M., Hosseinzadeh H. (2008). Synthesis of starch-poly (sodium acrylate-co-acrylamide) superabsorbent hydrogel with salt and pH responsiveness properties. Turk. J. Chem..

[14] Hsu S.C., Don T.M., Chiu W.Y. (2002). Free radical degradation of chitosan with potassium persulfate. Polym. Degrad. Stab..

